# Rationale Efficacy and Safety Evidence of Lenvatinib and Pembrolizumab Association in Anaplastic Thyroid Carcinoma

**DOI:** 10.3390/curroncol29100610

**Published:** 2022-10-14

**Authors:** Laurys Boudin, Jean-Baptiste Morvan, Juliette Thariat, Denis Métivier, Pierre-Yves Marcy, David Delarbre

**Affiliations:** 1 Department of Oncology, University Military Hospital Sainte-Anne, 2, Boulevard Sainte Anne, BP 600, 83000 Toulon, France; 2 Department of Head and Neck Surgery, University Military Hospital Sainte-Anne, 2, Boulevard Sainte Anne, BP 600, 83000 Toulon, France; 3 Department of Radiation Oncology, Centre François Baclesse, 14000 Caen, France; 4 Department of Nuclear Medicine, University Military Hospital Sainte-Anne, 2, Boulevard Sainte Anne, BP 600, 83000 Toulon, France; 5 Department of Radiodiagnostics and Interventional Imaging, Polyclinics ELSAN Group, PolyClinics Les Fleurs, Quartier Quiez, 83189 Ollioules, France; 6 Department of Internal Medicine, University Military Hospital Sainte-Anne, 2, Boulevard Sainte Anne, BP 600, 83000 Toulon, France

**Keywords:** anaplastic thyroid cell carcinoma, pembrolizumab, lenvatinib, treatment, rationale

## Abstract

Anaplastic thyroid carcinoma (ATC) are highly aggressive malignant tumors with poor overall prognosis despite multimodal therapy. As ATC are extremely rare, no randomized controlled study has been published for metastatic disease. Thyrosine kinase inhibitors, especially lenvatinib and immune checkpoint inhibitors such as pembrolizumab, are emerging drugs for ATC. Few studies have reported the efficacity of pembrolizumab and lenvatinib association, resulting in its frequent off-label use. In this review, we discuss rationale efficacy and safety evidence for the association of lenvatinib and pembrolizumab in ATC. First, we discuss preclinical rationale for pembrolizumab monotherapy, lenvatinib monotherapy and synergistic action of pembrolizumab and lenvatinib in the metastatic setting. We also discuss clinical evidence for immunotherapy and pembrolizumab in ATC through the analysis of studies evaluating immunotherapy, lenvatinib and pembrolizumab lenvatinib association in ATC. In addition, we discuss the safety of this association and potential predictive biomarkers of efficiency.

## 1. Introduction

Thyroid cancer includes a broad spectrum of histological tumor types and varies from indolent microscopic disease to highly aggressive dedifferentiated tumors [1]. Anaplastic thyroid carcinoma (ATC) comprises approximatively 1% of them and is characterized by rapid growth with local and distant evolution [2]. In the revised 8th edition of the TNM classification, all ATC patients are classified as stage IV and patients with distant metastasis are classified as stage IV C [3]. For the non-metastatic disease, current first-line treatment recommendations are based on surgery radiotherapy and targeted therapies, depending on the presence of the BRAF V600E mutation [4]. Despite this multimodal approach, the prognosis in patients with ATC is poor, with a median overall survival (OS) of 9.5 months [5]. In the metastatic setting, treatment options are limited to systemic treatments and supportive care. Palliation of locoregional disease may be used occasionally to alleviate compressive symptoms and pain in the short term.

Lenvatinib is an antiangiogenic (VEGFR 1–3/FGFR1–4) and antiproliferative (RET/PDGFR) tyrosine kinase inhibitor (TKI), which is approved for differentiated thyroid carcinoma (DTC) refractory to radioiodine treatment [6]. Progression free survival (PFS) for poorly DTC (PDTC) is only 14.8 months and even shorter in ATC [7]. All patients treated with lenvatinib monotherapy ultimately develop treatment resistance and progression [8].

Pembrolizumab is an immune checkpoint inhibitor targeting programmed cell death protein 1 (PD-1) on immune cells. The response to pembrolizumab immunotherapy is associated with the elevated expression of programmed death-ligand 1 (PD-L1) or high tumor mutational burden (TMB) [9,10,11]. In ATC associated with high PD-L1/TMB, the effect of immune checkpoint inhibitors is still low, with 1.9 months of median progression-free survival and 4.4 months of median overall survival (OS) [12]. Rapid tumor development is incompatible with a low response rate and/or a long time to response [13].

The combination of lenvatinib and pembrolizumab is based on a strong mechanistic rationale with immunomodulatory properties: increased tumor infiltration of effector CD8^+^ T cells and decreased monocytes and macrophages [14]. Adding lenvatinib to immune checkpoint inhibitors (ICIs) may help to overcome primary and acquired resistance to immunotherapy [15]. This combination shows survival benefits in phase-III studies for advanced endometrial cancer after the failure of platinum-based chemotherapy [16] and advanced renal cell carcinoma [17].

The fact that ATC is an extremely rare cancer makes it difficult to carry out phase-III studies, especially in metastatic setting. However, there is a rationale for the association of pembrolizumab and lenvatinib in this population. Some clinical data reported its efficacy, resulting in its frequent off-label use [18]. Our review article summarizes the rationale, efficacy, and safety evidence for the association of lenvatinib and pembrolizumab in anaplastic thyroid carcinoma.

## 2. Methods

A non-systematic literature search was conducted on PubMed using the terms ‘anaplastic thyroid carcinoma’, ‘thyroid carcinoma’, ‘immunotherapy’, ‘pembrolizumab’, ‘tyrosine kinase inhibitors’, and ‘lenvatinib’. The same search terms were used for the ClinicalTrials.gov registry of clinical trials. Review papers and clinical trials were retrieved and published between the years 2015 and 2022. Additional articles were retrieved based on the content of the initial articles reviewed, and additional searches were conducted, including the search terms ‘Tumor Mutation burden’, ‘PDL-1’, ‘TPS’, ‘FGFR’, ‘Microsatellite instability’, ‘TMB’, and ‘MSI’, ‘Tumor-Associated Macrophages’ in combination with ‘thyroid carcinoma’ and/or ‘predictive biomarkers’. Only English studies were included.

## 3. Preclinical Rationale

### 3.1. Preclinical Rationale of Pembrolizumab in Anaplastic Thyroid Carcinoma

The rationale for immunotherapy in ATC is based on the tumor microenvironment, tumor mutation burden (TMB) and Microsatellite instability.

Immune infiltrate Tumor-Associated Macrophages (TAMs), which can make up as much as 70% of the total tumor mass and may function as an immunosuppressive tumor stroma, are a key component of ATC tumors [19,20].

Elevated TAM levels in ATC tumor samples cause a hot immunological environment in 34% of cases, which is characterized by high expression of various inhibitory immune checkpoint mediators, including programmed death-ligand 1 (PD-L1) [4,19]. In both a phase-I investigation of the immune checkpoint inhibitor spartalizumab [20] and a pre-clinical study [21], 70% of ATC samples had PD-L1 expression. In the five cohort publications analyzing PD-L1 expression in ATC, PD-L1 positivity seemed higher in ATC than in DTC or PDTC [22,23,24,25,26]. In addition, CD274 gene amplification is present in 5.1% of ATCs, which is among the ten highest rates of all cancers [27,28]. PD-L1 is a potential predictive biomarker of immunotherapy response in ATC. Spartalizumab, a PD-1 inhibitor, produced an objective response in a recently released ATC cohort from a phase-I/II trial only in patients with detectable PD-L1 expression [20]. The tumor proportion score (TPS) is a PD-L1 measurement in which only membranous staining of tumor cells is regarded as a significant staining. In a recent multicenter research, TPS was detected at 5% in 73% of ATC samples and at least 1% in all ATC [29]. The TPS is a known pembrolizumab response marker established notably for non-small-cell lung cancer and potentially extended to other tumors such as squamous cell carcinoma of head and neck and ATC [30].

Tumor mutational burden (TMB), defined as the total number of somatic mutations per coding area of a tumor genome, is a measure of all non-synonymous coding mutations in a tumor exome [31]. TMB has been shown to vary widely from patient to patient and across tumor types. In striking contrast to PTC, the cancer genome of ATC displays the consequences of genomic instability and is characterized by a significantly higher mutational burden [32]. Comparisons between ATC TMB and TMB in DTCs and PDTCs were analyzed by Pozdeyev et al. using next-generation sequencing (NGS) gene panels in two cohorts of tumor samples (DTC *n* = 583 and ATC *n* = 196). Genetic changes were much more prevalent in ATCs than DTCs [33]. Landa et al. sequenced 117 thyroid cancers using NGS including 84 PDTCs and 33 ATCs. Compared to PDTCs, ATCs showed noticeably more mutations [34]. Similarly, 113 tumor samples from DTC, PDTC, and ATC were included in another cohort for TMB analysis, and the results showed that TMB in ATC was greater than in the other subtypes [35]. Several analyses have shown a correlation between high TMB (measured with various methods and cutoff points across studies) and the clinical benefit of immune checkpoint inhibitors [36]. In patients with non-small-cell lung cancer (NSCLC) treated with pembrolizumab as monotherapy, high tissue TMB were associated with durable clinical benefit and extended progression-free survival [37]. A recent biomarker analysis of multi-tumor KEYNOTE-158 study showed that TMB-high status identifies a subgroup of patients who could have a robust tumor response to pembrolizumab monotherapy [11]. TMB could be a novel and useful predictive biomarker for response to pembrolizumab monotherapy in patients with previously treated recurrent or advanced or metastatic solid tumors such as ATC.

Microsatellite instability is caused by the inactivation of the DNA mismatch repair gene(s) encoding mismatch repair (MMR) enzymes MLH1, MLH3, PMS1, and PMS2. This inactivation is a result of ongoing oxidative stress, which also damages the genome and causes poor DNA repair [38]. A genetic analysis of 779 advanced differentiated and anaplastic thyroid cancers noted the presence of MMR DNA deficiency in up to 46% of thyroid cancers with high mutational burden, especially ATCs [33]. A recent literature review revealed the prevalence of microsatellite instability in 7.4% of ATC, with mutations in the MSH2 gene (33%) being the most frequent, followed by MSH6 (25%) and MLH1 (16.7%) occurring in the following combinations: MLH1-MSH2 (8.3%), MSH2-MSH6 (8.3%), and MLH3-MSH5 (8.3%) [39]. In addition, Yoo et al. analyzed somatic copy-number changes in a thyroid tumor samples and showed that they were more common in ATC than in DTC, suggesting that ATC may have a more unstable genome than DTC [35]. Microsatellite instability due to defects in DNA mismatch repair proteins leads to an increase of mutation burdens in cancer-related genes and the formation of neoantigens, which activate the host’s anti-tumor immune response [40,41,42,43]. In cancers treated with immunological check-point inhibitors, mismatch repair deficit or high microsatellite instability has been shown to be strongly correlated with long-term immunotherapy-related responses and a better prognosis in several clinical trials. Pembrolizumab has currently been approved for metastatic tumors with high microsatellite instability or mismatch repair deficiency including ATC [44].

### 3.2. Preclinical Rationale of Lenvatinib in Anaplastic Thyroid Carcinoma

Lenvatinib is an oral multi-kinase inhibitor (VEGFR1–3, PDGFR, FGFR1–4, RET, and c-KIT) [8]. Several genetic alterations have been identified in ATC molecular pathways, involving VEGFR1, VEGFR2, EGFR, PDGFRα, and KIT that lead to tumor aggressiveness and progression [45,46]. Ferrari et al. demonstrated that lenvatinib inhibited primary ATC cell cultures proliferation in vitro, while also increasing apoptosis and inhibiting migration and invasion. The antiproliferative effect of lenvatinib in primary ATC cells was observed in all the samples, independently of the absence or presence of BRAF V600E mutation [47]. These outcomes were in accordance with other preclinical studies and showed lenvatinib could prevent angiogenesis by reducing vascular permeability and suppress tumor development in vivo in ATC cell lines [48,49].

RAS-RAF-MAPK, ERK and PI3K pathways are involved in the carcinogenesis of thyroid cancers [50,51] and mutations in these genes are present in ATC [52]. The proteins ERK and AKT were phosphorylated and activated in ATC, making them potential therapeutic targets. Lenvatinib reduced ERK1/2 and AKT phosphorylation in ATC cells, according to research by Ferrari et al. [47].

Lenvatinib also showed evidence of lowering EGFR phosphorylation and inhibiting cell growth via downregulating cyclin D1 expression [53]. Di Desidero et al. demonstrated that Sunitinib, another tyrosine kinase inhibitor (TKI), inhibits Akt and ERK1/2 phosphorylation and down-regulates cyclin-D1 to exert its anti-activated endothelium and ATC cell activity, both in vitro and in vivo [54]. In addition, 67% of ATCs and 77% of ATCs, respectively, were found to express cyclin D1 in studies by Lee et al. [55] and Wiseman et al. [56].

Although many of the TKIs used to treat thyroid cancers that are resistant to radioiodine have similar properties, such as antiangiogenic TKI action, it has been hypothesized that lenvatininb’s greater clinical response may be due to its capacity to also target FGFR1–4 in these rare thyroid tumors [57]. In comparison to normal thyroid and DTC samples, PDTC and ATC samples had the greatest levels of FGFR1 expression. Immunohistochemistry was used by Yamazaki et colleagues to examine FGFR4 expression in 12 ATC patients, and they hypothesized that FGFR4 expression would predict how well lenvatinib would work [58]. Adam and colleagues discovered considerably greater expression of the FGFR1–4 combo in ATC compared to normal thyroid using RNAscope in-situ hybridization [29].

### 3.3. Preclinical Rationale of Pembrolizuman and Lenvatinib Association in Anaplastic Thyroid Carcinoma

**Figure 1:**
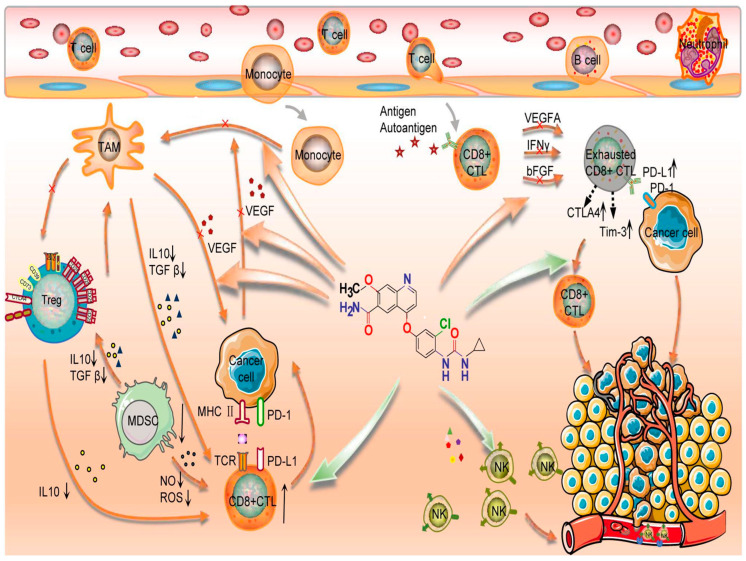
Lenvatinib’s action mode when combined with immunological check point inhibitors. TAM: Tumor associated macrophages; Treg: Regulatory T cells; IL: Interleukine; TGF: transforming growth factor; M DSC: Myeloid-derived suppressor cells; MHC: major histocompatibility complex; IFN: Interferon; ROS: Reactive oxygen species; NO: nitric oxyde; FGF: fibroblast growth factor; CTL: cytotoxic T lymphocyte; VEGF: Vascular Endothelial Growth Factor; NK: Natural killer; TCR: T cel receptor; Tim-3: T-cell immunoglobulin and mucin containing protein-3; PD-1: Programmed cell death protein 1; PD-L1: Programmed death-ligand 1; CTLA 4: Cytotoxique-T-Lymphocyte-Antigen 4 protein; Green arrow: Activation signal; Red arrow: Inhibition signal. Star: Antigen. Cross: Inhibition.

VEGF-targeted therapies may also function, in part, by blocking VEGF-mediated immune suppression in addition to their anticancer and antiangiogenic effects [59]. In six distinct malignancies, including thyroid carcinomas, immune checkpoint inhibitors and lenvatinib target genes were significantly upregulated and displayed driving alterations, according to a multi-omics investigation [60]. Pathway-enrichment analysis found target genes were implicated in tumor development, angiogenesis, and immunoregulatory associated pathways. These findings are resumed in Figure 1 [60]. Otherwise, lenvatinib and monoclonal antibodies against PD-1 enhanced the immune response in syngeneic mice models. Kato et al. investigated the immunomodulatory activities of lenvatinib in the tumor microenvironment and its mechanisms of enhanced antitumor activity when combined with PD-1 blockade. The antitumor activity of lenvatinib plus anti-PD-1 was greater than that of either single treatment. Flow cytometric analysis revealed that lenvatinib reduced tumor-associated macrophages (TAMs) and increased the percentage of activated CD8^+^ T cells secreting interferon-γ^+^ and granzyme B. Combination treatment further increased the percentage of T cells, especially CD8^+^ T cells, among CD45^+^ cells and increased interferon-γ^+^ and granzyme B CD8^+^ T cells. Transcriptome analyses of tumors resected from treated mice showed that genes specifically regulated by the combination were significantly enriched for type-I interferon signaling [61]. Lenvatinib’s anticancer and immunomodulatory properties were studied by Kimura et al. with a syngeneic hepatocarcinoma mouse tumor model. They showed that lenvatinib had more antitumor activity than sorafenib in a Hepa1-6 tumor model using immunocompetent mice. In addition, CD8^+^ T cell depletion significantly reduced the antitumor activity of lenvatinib but not sorafenib, indicating that lenvatinib exhibited immunomodulatory activity, particularly on the CD8^+^ T cell population, and that this effect contributed to lenvatinib’s potent antitumor activity under the immunocompetent condition [14]. Lenvatinib therapy has also been demonstrated to suppress TH2 and boost TH1 immunological responses, by activating memory T cells [62]. Gunda et al. provided evidence for the justification for combining PD-L1 and lenvatinib in ATC using an ATC immunocompetent mice model [63]: lenvatinib caused a noticeable rise in tumor-associated macrophages and tumor-infiltrating immune cells, as well as a noticeable rise in peripheral and tumoral myeloid derived suppressor cells (PMN-MDSC), which together showed dramatic alterations in the immune microenvironment. They came to the conclusion that lenvatinib exhibited pro- and anti-inflammatory effects on the immune system. By reducing the amount of immunosuppressive PMN-MDSC in an experimental manner, they were able to show better antitumoral activity. Inhibition of the PD-1/PD-L1 axis was also associated with a decrease in some immunosuppressive cell types. Thus, lenvatinib plus immune check point inhibitors act synergistically to block PD1/PDL1 axis and the formation of an immunosuppressive microenvironment [64]. Adam et al. looked at the possible immunostimulatory function of FGFR1–4, another Lenvatinib target, in ATC tumor cells. They discovered that the combination of FGFR1–4 was substantially more expressed in ATC than in normal thyroid, but that leukocytes infiltrating the tumor did not express FGFR1–4 [29]. Taken together, these preclinical studies demonstrating lenvatinib’s immunomodulatory activity provide mechanistic rationale for the study of lenvatinib in combination with an anti-PD-1 agent such as pembrolizumab in ATC.

This figure details mechanisms of immunological checkpoint inhibitors combined with lenvatinib. Lenvatinib increased the CD8+T cells function and the cytotoxicity of NK cells, decreased the expression of PD-1, CTLA-4, and TIM3 in T cells, and inhibited T cell exhaustion. Lenvatinib also inhibits tumour angiogenesis and abnormalities by inhibiting the secretion of angiogenic factors, such as VEGF, FGF, and PDGF. Finally, immune checkpoint inhibitors restore the exhausted T cell activity to kill the cancer cell [60].

## 4. Clinical Evidence

### 4.1. Clinical Evidence of Immunotherapy Alone in ATC

Four studies evaluating immunotherapy alone have been published in thyroid cancers [12,20,65,66]. Considering the preclinical rationale of ICI in TCAs, three of them included TCAs [12,20,65]. In Table 1, the results of these clinical studies are summarized.

In a phase-I/II clinical trial for patients with locally advanced and/or metastatic ATC, the effectiveness of spartalizumab PD-1 inhibitor was evaluated [20]. Phase II of the study’s findings have been published: 42 patients were enrolled and an overall response rate (ORR) of 19% (including 7% CR) was achieved. Median PFS was 1.7 months. Median OS was 5.9 months with a one-year survival rate of 40%. BRAF wild-type tumors were found in all patients who had a complete response (CR). Biomarker analysis was performed: 70% of patients exceeded the PD-L1 positivity criterion (1% positive in tumor cells), and positivity was linked with statistically significant variations in ORR. Patients with 1% CD8 baseline expression had a statistically greater ORR. Finally, baseline IFN- signatures were acquired from 18 patients using RNA sequencing. The best percentage change of target lesions by RECIST criteria and IFN- signature were shown to be correlated [20].

Concerning pembrolizumab, Hatashima et al. recently published a retrospective case series of 13 patients with locally advanced or metastatic unresectable ATC who received immune checkpoint inhibitor therapy (pembrolizumab or nivolumab) at a single institution [12]. In this study, the objective response rate was 16%. Only two patients had a partial response, and three patients had durable stable disease. Median OS was 4.4 months and one-year survival rate 38%. Among patients with a clinical benefit, after a median follow-up of 13.5 months, median OS had not been reached with a patient alive after 29 months [12]. Concerning safety, 46% of patients experienced immune-related adverse events in this study. Grade 3 and above adverse events were noted in two patients (15%) [12].

Only one study explored the effectiveness of a combination of immune checkpoint inhibitors. This phase-II trial analyzed the association of nivolumab and ipilimumab in three distinct cohorts: DTC with radioiodine resistance, locally advanced or metastatic ATC and metastatic MTC. The response rate was 30% for the seven patients of the ATC cohort. Unfortunately, median OS, median PFS and one-year survival rate were not published [65].

These studies highlight that ATC patients treated with immune checkpoint inhibitor therapy have a particularly unfavorable prognosis. Despite low response rates, immune checkpoint inhibitors such as pembrolizumab may be a treatment option for a subset of patients. This hypothesis is supported by case reports reporting extended response with pembrolizumab in ATC. Nabhan et al. reported a patient of unresectable treatment-naïve ATC showing a dramatic and durable response to first-line pembrolizumab therapy. Eighteen months after digagnosis, the patient was still alive with no evidence of disease progression [67]. A case of a patient with ATC for whom pembrolizumab was used after chemotherapy and dabrafenib/trametinib (selective BRAF and MEK inhibitor, respectively) was also reported. The patient achieved a partial response to therapy, enabling a complete surgical resection followed by postoperative chemoradiation. The patient remained alive one year after treatment [68]. The example of a patient with constricted airway and esophagus upon diagnosis was published by Spalart et al. The patient had a quick and profound response to first-line pembrolizumab with exclusive brain progression after 18 months of treatment [69].

In addition to current clinical trials in ATC testing pembrolizumab (NCT05119296) and atezolizumab (NCT03181100), a study evaluating a dual PD-1 and CTLA-4 inhibitor is ongoing (NCT05453799).

### 4.2. Clinical Evidence of Lenvatinib Alone in ATC

Thirteen studies evaluated efficacy and safety of lenvatinib alone in ATC: four single-arm phase-II studies [70,71,72,73], eight retrospective studies [58,74,75,76,77,78,79,80] and a meta-analysis [81]. All these studies are reported in Table 2.

In 2017, Tahara and collaborators published in a phase-II study evaluating in patients with thyroid cancer in which 17 patients with ATC were enrolled. The median PFS was 7.4 months, the median OS was 10.6 months and the objective response rate was 24% [73].

Higashiyama et al. published the largest and the most recent prospective phase-II study [70]. In this multicenter study, 52 patients were enrolled from 17 institutions. Forty-two were included for efficacy analysis, and 50 patients were included for safety analysis. The estimated 1-year overall survival rate was 11.9% (95% CI, 4.4–23.6%). One patient (2.4%) achieved complete response, four patients (9.5%) partial response, and 26 patients (61.9%) stable disease, including nine patients (21.4%) who demonstrated durable stable disease, giving an objective response rate of 11.9%, disease control rate of 73.8%, and clinical benefit rate of 33.3%. Adverse events of any grade were observed in 45 patients (90.0%), the most common of which of any grade included loss of appetite (48.0%), fatigue (48.0%), hypertension (44.0%), and palmar-plantar erythrodysesthesia syndrome (26.0%). Eight patients (16.0%) discontinued lenvatinib due to intolerable adverse events, as deemed by the investigator. Median dose intensity was 19.84 mg/day. Thirty-one patients (62.0%) required a dose reduction, and median time to first dose reduction was 11 days [70].

A newly published meta-analysis [81] also includes more research. The combined findings revealed that the pooled overall response rate and disease control rate were, respectively, 15.0% and 63.0%. Median PFS and OS were both 3 months. The 12-month PFS rate was 8.4%, whereas the 12-month OS rate was 18.9%. Hypertension (56.6% of AEs), proteinuria (32.6% of AEs), and fatigue (32% of AEs) were the main toxicities of lentivatinib [81]. The most frequent adverse event (AE) in the meta-analysis was hypertension, which was effectively managed by adjusting the dose and giving antihypertensive medication. Proteinuria can be treated with lenvatinib dose reduction and timely withdrawal to prevent renal failure [8,76]. It is still unclear whether lenvatinib played a role in the deaths of two patients who had pneumothorax-related AE and three patients who had severe hemoptysis in the pooled meta-analysis [81]. The risk of bleeding exists in lesions close to large blood vessels, necessitating careful management [82]. Particularly vulnerable to vessel wall rupture are lesions with a history of external irradiation [83] and fistulae created in the skin or digestive tract [84]. Although it is an uncommon side effect, pneumothorax that develops while receiving lenvatinib for thyroid cancer has already been reported to be fatal [85].

Altogether, these studies highlight that lenvatinib’s effect on extending survival in ATC is not statistically significant. Lenvatinib had a significant antitumor effect in ATC patients compared to other multikinase inhibitors of VEGF receptors, such as pazopanib and sorafenib, which were used as monotherapy for [86,87]. A phase-II study evaluating lenvatinib as neoadjuvant therapy in locally advanced invasive thyroid cancer is ongoing (NCT04321954).

### 4.3. Clinical Evidence of Lenvatinib and Pembrolizumab Association in ATC

Considering lenvatinib antitumor activity and extended response with pembrolizumab in a subset of patient with ATC, recent studies evaluated their association. Three studies (two retrospective [13,88] and one prospective [89]) and five case reports [18,90,91] have been published so far. Study results are resumed in Table 3, and case reports results are reported in Table 4.

In a retrospective study [13], Dierks et al. analyzed six patients with metastatic ATC and two patients with PDTC, who received a combination therapy of lenvatinib and pembrolizumab. Lenvatinib was started at 14–24 mg daily and combined with pembrolizumab at a fixed dose of 200 mg every three weeks. This study demonstrated 66% of CR, 16% of partial response and 16% of stable disease SD. With treatment periods ranging from 1 to 40 months, the median progression-free survival for ATCs was 16.5 months. Four patients experienced grade III/IV toxicity, necessitating lenvatinib dose modification or interruption. The median OS was 18.5 months, and despite metastatic disease three ATC patients are still alive without recurrence after 40, 27, and 19 months. All of the patients in this cohort who had long-term (>2 years) responses had either a higher TMB or a PD-L1 TPS higher than 50%. After these results Dierks et al. analyzed the combination treatment in the ATLEP phase-II clinical trial [89].

Only one prospective phase-II study evaluated the combination of pembrolizumab and lenvatinib in patients with ATC [89]. Thirty-two patients without BRAF V600E mutation were included with three different doses of lenvatinib: 20, 14, and 10 mg. Final results for 27 ATC patients demonstrated an overall respose rate and clinical benefits rate at 2 years of 51.9% and 96.3%, respectively. Median PFS and OS were respectively evaluated at 10 months and 11 months with 26% of patients who survived more than two years. Concerning safety, 53% patients experienced Grade 3 or 4 adverse events including fistula, bleeding and sepsis [89].

Lyer et al. analyzed pembrolizumab and lenvatinib combination using a different time-scale. Pembrolizumab was added to kinase inhibitors at progression of ATC. For the five patients for whom pembrolizumab was added to lenvatinib, ORR was 40% and a one-year survival rate was 40% [88].

Four out of the five case reports published report a partial response with pembrolizumab and levantinib association with survival from 1 to more than 18 months [18,90,91]. One patient demonstrated pure red cell aplasia with pembrolizumab [18] and in another patient under pembrolizumab and lenvatinib, grade 4 transaminitis occurred [91].

These studies support better efficacy of the combination than pembrolizumab or lenvatinib as monotherapy in locally advanced or metastatic ATC. Safety results are consistent with the phase-III Study KEYNOTE-775, which evaluated lenvatinib plus pembrolizumab versus chemotherapy in patients with advanced endometrial cancer and prior treatment with platinum-based chemotherapy regimen. In this larger study, Grade 3 or higher adverse events occurred in 88.9% of the patients receiving lenvatinib plus pembrolizumab and the most frequent any-grade treatment-emergent adverse events were hypertension (64.0%), hypothyroidism (57.4%) and diarrhea (54.2%) [16,92].

A phase-II study evaluating lenvatinib and pembrolizumab for the treatment of stage IVB and IVC anaplastic thyroid cancer is recruiting (NCT04171622). In this study, DNA changes and immune biomarkers will be examined.

## 5. Conclusions

Tumor microenvironment, tumor mutation burden and microsatellite instability associated with ATC provide a strong rationale for ICIs treatment such as pembrolizumab. Unfortunately, studies evaluating immunotherapy alone in ATC are disappointing. Lenvatinib demonstrated strong antitumor activity but a non-significant effect on prolonging survival in ATC. Nevertheless, lenvatinib’s immunomodulatory activity provides mechanistic rationale for a lenvatinib combination with anti-PD-1. Literature data are in favor of a synergistic action of the combination allowing response rate never achieved in the metastatic population as well as long survival for some patients. TMB or a PD-L1 TPS > 50% are potential efficacy biomarkers. Despite frequent grade 3 or 4 AEs, the safety of this combination seems manageable.

In summary, pembrolizumab and lenvatinib association offers a new therapeutic opportunity for metastatic ATC, which is a very poor prognosis cancer. Although ATCs are very rare, clinical studies are urgently warranted to assess the place of this combination in the therapeutic sequence and identify efficacy predictive biomarkers for this treatment.

## Figures and Tables

**Table 1:** Studies evaluating immunotherapy in anaplastic thyroid cell carcinoma.

Study	ICI	Nb of Patient Included	Median Age	Previous Systemic Treatment (%)	ORR	Median PFS (Months)	Median OS (Months)	One Year Survival Rate
Hatashima 2022 [12]	Pembrolizumab (12 patients). Nivolumab (1 patient)	13	70	23%	16%	1.9	4.4	38%
Capdevila 2020 [20]	Spartalizumab	42	62	40%	19%	1.7	5.9	40%
Lorch 2020 [65]	Ipilimumab + Nivolumab	10	65	NA	30%	NA	NA	NA

Nb: Number; NA: Not available. ICI: Immune check point inhibitors. ORR: overall response rate. PFS: Progression free survival. OS: overall survival.

**Table 2:** Studies evaluating lenvatinib in anaplastic thyroïd cell carcinoma.

Study References	Methodology	Nb of Patient Included	Median Age (Years)	Previous Systemic Treatment Rate	ORR	Median PFS (Months)	Median OS (Months)	One-Year Survival Rate
Huang 2022 [81]	Meta-analysis	176	NA	NA	15%	3.1	3.2	18.9%
Higashiyama 2022 [70]	Prospective	42	73	60%	12%	NA	5.0	11.9%
Tahara 2017 [73]	Prospective	17	65	59%	24	7.4	10.6	NA
Wirth 2021 [71]	Prospective	34	NA	70%	3%	2.6	3.2	28%
Takahashi 2019 [72]	Prospective	17	65	NA	24%	7.4	10.6	NA
Fukuda 2020 [74]	Retrospective	13	68	69%	23%	3.8	10.2	NA
Ishihara 2021 [75]	Retrospective	10	69	50%	30%	NA	4.7	15%
Iwasaki 2021 [76]	Retrospective	32	77	NA	19%	NA	3.2	NA
Iyer 2018 [77]	Retrospective	10	67	NA	30%	2.6	3.9	NA
Kim 2020 [78]	Retrospective	14	66	NA	29%	5.7	6.7	NA
Park 2021 [79]	Retrospective	11	NA	NA	27%	NA	NA	NA
Sparano 2021 [80]	Retrospective	15	67	93%	0%	NA	2.7	NA
Yamazaki 2020 [58]	Retrospective	20	74	NA	10%	NA	NA	NA

Nb: Number; NA: Not available. ICI: Immune check point inhibitors. ORR: overall response rate. PFS: Progression free survival. OS: overall survival.

**Table 3:** Studies evaluating pembrolizumab and lenvatinib association in anaplastic thyroid cell carcinoma.

References	Methodology	Nb of Patient Included	Median Age (Years)	Previous Systemic Treatment	ORR	Median PFS (Months)	Median OS (Months)	One Year Survival Rate
Dierks 2021 [13]	Retrospective	6	NA	83%	66%	17.7	18.5	50%
Dierks 2022 [89]	Prospective	29	NA	NA	52%	10	11	NA
Iyer 2018 [88]	Retrospective	5	60	60%	60%	8.3	8.3	40%

Nb: Number; NA: Not available; ORR: overall response rate; PFS: Progression free survival; OS: overall survival.

**Table 4:** Cases report of pembrolizumab and lenvatinib association in anaplastic thyroid cell carcinoma.

References	Age (Years)	Previous Systemic Treatment	Follow Up	Best Response	Duration of Treatment Response (Months)	OS (Months)
Shih 2022 [90]	71	No	1 months	PD	1	1
Shih 2022 [90]	58	Yes	2.7 months	PR	2.7	2.7
McCrary 2022 [18]	54	Yes	NA	PR	NA	NA
McCrary 2022 [18]	59	No	3 months	PR	NA	NA
Luongo 2021 [91]	54	Yes	18 months	PR	18	18

PD: Progression disease; PR: Partial response; OS: overall survival; NA: Not available.

## Data Availability

Not applicable.
